# Longitudinal Grey and White Matter Changes in Frontotemporal Dementia and Alzheimer’s Disease

**DOI:** 10.1371/journal.pone.0090814

**Published:** 2014-03-03

**Authors:** Lars Frings, Belinda Yew, Emma Flanagan, Bonnie Y. K. Lam, Michael Hüll, Hans-Jürgen Huppertz, John R. Hodges, Michael Hornberger

**Affiliations:** 1 Center of Geriatrics and Gerontology, University Medical Center, Freiburg, Germany; 2 Department of Nuclear Medicine, University Medical Center, Freiburg, Germany; 3 Neuroscience Research Australia, Sydney, Australia; 4 Swiss Epilepsy Centre, Zürich, Switzerland; 5 ARC Centre of Excellence in Cognition and its Disorders, Sydney, Australia; 6 School of Medical Sciences, University of New South Wales, Sydney, Australia; University of Jaén, Spain

## Abstract

Behavioural variant frontotemporal dementia (bvFTD) and Alzheimer’s disease (AD) dementia are characterised by progressive brain atrophy. Longitudinal MRI volumetry may help to characterise ongoing structural degeneration and support the differential diagnosis of dementia subtypes. Automated, observer-independent atlas-based MRI volumetry was applied to analyse 102 MRI data sets from 15 bvFTD, 14 AD, and 10 healthy elderly control participants with consecutive scans over at least 12 months. Anatomically defined targets were chosen a priori as brain structures of interest. Groups were compared regarding volumes at clinic presentation and annual change rates. Baseline volumes, especially of grey matter compartments, were significantly reduced in bvFTD and AD patients. Grey matter volumes of the caudate and the gyrus rectus were significantly smaller in bvFTD than AD. The bvFTD group could be separated from AD on the basis of caudate volume with high accuracy (79% cases correct). Annual volume decline was markedly larger in bvFTD and AD than controls, predominantly in white matter of temporal structures. Decline in grey matter volume of the lateral orbitofrontal gyrus separated bvFTD from AD and controls. Automated longitudinal MRI volumetry discriminates bvFTD from AD. In particular, greater reduction of orbitofrontal grey matter and temporal white matter structures after 12 months is indicative of bvFTD.

## Introduction

Behavioural variant frontotemporal dementia (bvFTD) and Alzheimer’s disease (AD) are the two most prevalent early-onset dementias [1]. Diagnostic criteria for both have been proposed [2], [3] but clinical diagnosis remains challenging [4], [5].

The revised diagnostic criteria for bvFTD [6] incorporate neuroimaging evidence of frontal and/or temporal brain atrophy changes at presentation. AD patients also show atrophy compared to healthy controls particularly in the medial temporal lobe [7], as well as precuneus [8] at presentation. By contrast, bvFTD show characteristic frontal and anterior temporal lobe atrophy [9]–[11], with the hippocampus being affected to a similar degree as in AD [12]–[14]. Similarly, bvFTD and AD show differential white matter changes involving frontal and parietal structures, respectively, while both groups appear to show similar levels of temporal lobe white matter change [12]–[14].

Despite the identification of typical atrophy patterns in at clinic presentation in bvFTD and AD, longitudinal grey and white matter changes have not been reported yet might be particularly relevant for aiding diagnosis and for measuring disease progression in context of potential disease modifying therapies.

The current study set out to investigate these longitudinal changes in bvFTD and AD, compared to age-matched controls via a novel automated, observer-independent method of atlas-based MRI volumetry. This technique allows establishing volumetrics both at cross-sectional and longitudinal levels. We hypothesised that bvFTD and AD will show cross-sectionally the previously described patterns of atrophy, while the longitudinal changes would reveal additional regions affected in both diseases.

## Methods

### Ethics statement

The study was conducted in compliance with the Declaration of Helsinki (1991). Data collection for this study had been approved by the local ethics committees (University Medical Center, Freiburg; South Eastern Sydney and Illawarra Area Health Service and the University of New South Wales).

### Case selection

Thirty-nine participants were selected from the FRONTIER database, Sydney, Australia and from a project database (German Federal Ministry of Education and Research project ‘Mechanisms of brain reorganisation in the language network’, BMBF: 01GW0662) in Freiburg, Germany. Data from 10 bvFTD and 14 AD patients, as well as 10 controls have been acquired in Sydney. The study sample was complemented by 5 bvFTD patients from Freiburg. Participants in the control group had been chosen to be not significantly different from patient groups in terms of age and education. In total, 102 MRI data sets from these participants were analysed. All bvFTD patients met current consensus criteria for FTD [6], [15] with insidious onset, decline in social behaviour and personal conduct, emotional blunting and loss of insight. Of the 10 bvFTD patients from the FRONTIER database, 3 had the C9ORF72 genetic mutation. All AD patients met NINCDS-ADRDA diagnostic criteria [16] for probable AD, with episodic memory deficits being the predominant symptoms (see Table 1 for demographic details). Age- and education-matched healthy controls were selected from a healthy volunteer panel or were spouses/carers of patients. All patients gave written informed consent.

**Table 1:** Demographics, Cognitive & Behavioural Tests.

	bvFTD	AD	CON	ANOVA (Main Effect of Group)	bvFTD vs CON	AD vs CON	bvFTD vs AD
N	15	14	10				
Gender (M/F)	11/4	10/4	5/5	n.s. (chi-squared test)			
Age	61.6 (6.6)	63.9 (7.4)	65.5 (6.2)	n.s.			
Education	11.9 (2.2)	12.8 (3.5)	13.2 (1.8)	n.s.			
MMSE [30]	25.9 (2.7)	25.3 (2.8)	29.0 (1.2)	*	*	*	n.s.
FRS logit score^1^	0.1 (1.3)	1.5 (1.2)	-	*			
ACE-R [100]^2^	79.9 (7.5)	74.9 (10.4)	93.6 (4.7)	*	*	*	n.s.
CBI-R [180]^3^	59.3 (25.2)	35.7 (22.5)	4.6 (2.6)	*	*	*	*

n.s.  =  not significant; *  =  p0.05.

Data available from ^1^ 6 bvFTD and 11 AD, ^2^ 10 bvFTD, 14 AD, and 10 controls, ^3^ 10 bvFTD, 10 AD, and 9 controls. Maximum scores for tests given in brackets. Per group mean and S.D. (in parentheses).

All participants from the FRONTIER database underwent general cognitive screening using the Addenbrooke’s Cognitive Examination (ACE-R) [17] to determine their overall cognitive functioning. The ACE-R results in a score out of 100, and includes subsections in attention, memory, language and visuo-perception. The frontotemporal dementia rating scale (FRS) [18] was used to determine the disease severity in bvFTD and AD patients. The Cambridge Behavioural Inventory (CBI) was used as a behavioural disturbance measure with higher scores indicating more behavioural disturbance as reported by the family or carer. Participants from the Freiburg database were assessed using the MMSE and the Clinical Dementia Rating.

### MRI acquisition

All patient and controls from the FRONTIER database underwent the same imaging protocol with whole-brain T1-weighted images using a 3T Philips MRI scanner with standard quadrature head coil (8 channels). The 3D T1-weighted sequences were acquired as follows: coronal orientation, 1×1 mm^2^ in-plane resolution, slice thickness 1 mm, TR/TE  =  5.8/2.6 ms. Structural T1-weighted MRI data from the Freiburg participants were acquired on a 3T Siemens TIM-Trio scanner equipped with a 12-channel headcoil using a 3D-MPRAGE sequence in sagittal orientation with 1×1 mm^2^ in-plane resolution, slice thickness 1 mm, and TR/TE = 2200/2.15 ms. The scanning protocols of the two sites were held constant across subjects and over time. All patients were scanned annually after a baseline scan. Controls had a baseline scan as well as a follow-up scan after two years. Median number of scans per subject was 2 (Mean  =  2.6, S.D.  =  0.7, range 2 to 4 scans), the mean delay between the first and last scan was 23.3 months (S.D.  =  7.7, range 12 to 36.7 months).

### MRI data processing and volumetric analysis

The MRI data processing and volumetry have been described in detail elsewhere [19], [20]. The method is based on SPM5 (statistical parametric mapping software, Wellcome Trust Centre for Neuroimaging, London, UK; http://www.fil.ion.ucl.ac.uk/spm), and masks derived from a probabilistic brain atlas provided by the Laboratory of Neuroimaging (LONI) at the University of California, Los Angeles, CA (LONI Probabilistic Brain Atlas (LPBA40); http://www.loni.ucla.edu/Atlases). The analysis is fully automated by use of a MATLAB batch script and requires about 12 minutes per MRI scan on an Xeon 5620 2.4-GHz PC (Intel, Santa Clara, California), with 2 quad cores and MATLAB multithreaded computation-enabled. In short, each T1-weighted volume dataset was normalized to the standard brain of the Montreal Neurological Institute (MNI) included in the SPM5 distribution and segmented into different brain compartments, i.e., grey matter (GM), white matter (WM), and cerebrospinal fluid. This was done by using the ‘‘unified segmentation’’ tool of SPM5 with its default settings. The segmentation resulted in ‘‘modulated’’ and ‘‘unmodulated’’ images for the different tissue compartments. Modulation compensates for dilatation or shrinkage during spatial normalization and has the effect of preserving the total amount of signal from the respective tissue class in the normalized partitions [21]. To determine the volume of a specific brain structure of interest the corresponding binary mask derived from the LPBA40 atlas was multiplied with the modulated image of the desired tissue class. The values of all voxels in the resulting image were summed up and divided by 1,000 to get the volume of the investigated structure in milliliter units. Because of modulation of the tissue images, the effect of normalisation (i.e., extension or shrinkage of the investigated structure) was compensated for so that the computed volume represented the volume of the original structure in native space. Target structures (31 in total, see Table 2) were chosen a priori for analyses of group differences in volume and volume change over time. As the frontal and temporal lobes typically show atrophy in bvFTD, we included as regions of interest all structures of the LPBA40 atlas that belong to the frontal and the temporal lobes. Additionally, we took into account recent studies that described atrophy of the caudate and the insula in bvFTD [10]. For bilateral structures, the volumes of left and right were summed up. It has to be noted that the structure labelled as ‘hippocampus’ in the LPBA40 atlas apparently comprises hippocampus and amygdala. Each dataset was processed independently from other datasets with the same, fully automated protocol, regardless of representing a baseline or follow-up dataset. Processing of follow-up scans did not require coregistration to baseline scans.

**Table 2:** Anatomical Structures Selected for Volumetric Analyses, based on the LPBA40 atlas.

	FL_GM_ — Frontal Lobe		FL_WM_ — Frontal Lobe
	SFG_GM_ — Superior Frontal Gyrus		SFG_WM_ — Superior Frontal Gyrus
	MFG_GM_ — Middle Frontal Gyrus		MFG_WM_ — Middle Frontal Gyrus
	IFG_GM_ — Inferior Frontal Gyrus		IFG_WM_ — Inferior Frontal Gyrus
	PreG_GM_ — Precentral Gyrus		PreG_WM_ — Precentral Gyrus
	MOFG_GM_ — Middle Orbitofrontal Gyrus		MOFG_WM_ — Middle Orbitofrontal Gyrus
	LOFG_GM_ — Lateral Orbitofrontal Gyrus		LOFG_WM_ — Lateral Orbitofrontal Gyrus
	Gyrus Rectus_GM_		Gyrus Rectus_WM_
Grey Matter	TL_GM_ — Temporal Lobe	White Matter	TL_WM_ — Temporal Lobe
	STG_GM_ — Superior Temporal Gyrus		STG_WM_ — Superior Temporal Gyrus
	MTG_GM_ — Middle Temporal Gyrus		MTG_WM_ — Middle Temporal Gyrus
	ITG_GM_ — Inferior Temporal Gyrus		ITG_WM_ — Inferior Temporal Gyrus
	PHG_GM_ — Parahippocampal Gyrus		PHG_WM_ — Parahippocampal Gyrus
	FG_GM_ — Fusiform Gyrus		FG_WM_ — Fusiform Gyrus
	Hippocampus & Amygdala		
	Caudate		
	Insula		

### Statistical analysis

Data were analyzed using SPSS19.0 (SPSS Inc., Chicago, Ill., USA). Parametric demographic (age, education), neuropsychological (general cognitive tests), disease severity (FRS) and behavioural (CBI) data were compared across the three groups (bvFTD, AD and controls) via one-way ANOVAs. A chi-squared test was used to check for significant differences in gender across all groups. Results were regarded significant if p<0.05. Based on volumetric measures, for each of the target structures we calculated:

1. Individual volume at clinic presentation (in ml)

Volumes have been divided by individual intracranial volume (ICV) and multiplied by average ICV of controls, resulting in measures of normalised individual volume.

2. Annualised volume change (in %)

In order to take into account multiple measurements, individual change was approximated by a linear regression (with intercept) of volumes over time, with beta or slope representing change per year in ml. Change per year (in ml) was divided by volume at first presentation, resulting in annualised volume change (in %).

For group comparison statistics two separate sets of ANOVAs with fixed factor 'group' and post-hoc Bonferroni tests have been performed on (1) the independent variables 'volumes at clinic presentation', and (2) the independent variables 'annualised volume change' of all target structures (see Table 2). Results of the univariate volumetric analyses were regarded significant if p<0.05, corrected for multiple comparisons (variables). The analyses were done using the false discovery rate (FDR) in multiple testing under the assumption of mutual dependency of the analysed structures. Univariate tests were followed by correction of the original p-values according to the method of Benjamini and Hochberg [22] using the MATLAB script available at [www.mathworks.com/matlabcentral/fileexchange/27418-benjamini-hochbergyekutieli-procedure-for-controlling-false-discovery-rate]. This method controls for the expected proportion of false positive findings and it is less strict than Bonferroni correction. Results of the post-hoc tests were regarded significant if they survived an additional Bonferroni correction for multiple pairwise group comparisons.

The variables which showed a significant difference between bvFTD and AD in the former analyses were subjected to a binary logistic regression to determine the best predictor of diagnosis (bvFTD vs. AD).

## Results

### Demographics and global cognitive functioning

Demographics and general cognitive scores can be seen in Table 1. Participant groups did not differ in terms of age, education, or gender. However, the patient groups differed significantly in disease severity (Total FRS Corrected: *p*<0.05) with bvFTD patients being more impaired. For the cognitive screening tests (ACE-R and MMSE), both patient groups were significantly impaired in comparison to controls but did not differ from each other. Similarly, on the behavioural scores (CBI), bvFTD and AD patients showed significantly more behavioural disturbances than age-matched controls, and bvFTD displayed more severe impairment than AD patients (see Table 1).

### Brain volumes at clinic presentation

**Figure 1:**
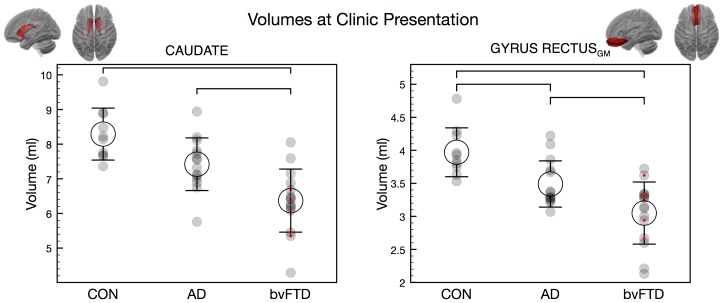
Volumes at clinic presentation (ml; Mean +/– 1 S.D.) of caudate and gyrus rectus grey matter. Brackets indicate significant group differences. Red dots indicate data from Freiburg. CON  =  control participants.

The univariate analyses revealed significant main effects of factor group for most grey matter volumes and some of the white matter volumes (see Table 3). Post-hoc Bonferroni tests revealed that the bvFTD group had significantly smaller volumes of all grey and white matter structures than the control group. The AD group, compared to controls, had reduced volumes of all grey matter structures, except for the middle frontal gyrus (MFG_GM_), middle orbitofrontal gyrus (MOFG_GM_), precentral gyrus (PreG_GM_) and the caudate. In contrast, for the white matter volumes only the middle temporal gyrus (MTG_WM_) was significantly reduced in AD compared to controls. bvFTD patients showed smaller volumes of the caudate and gyrus rectus_GM_ than AD (Table 3; Figure 1). At group level, AD patients did not have smaller volumes of any of the target structures than bvFTD patients.

**Table 3:** Anatomical structures that showed significant group differences of volumes at clinic presentation (FDR-corrected p0.05). Per group mean and S.D. (in ml), univariate between-subjects effects, and pairwise post-hoc Bonferroni test results.

Dependent Variable	bvFTD	AD	CON	F(2,36)	bvFTD vs CON	AD vs CON	bvFTD vs AD
**Grey Matter**							
Frontal Lobe							
SFG_GM_	44.75 (4.61)	48.8 (4.99)	56.64 (4.52)	19.07	*	*	n.s.
LOFG_GM_	4.38 (0.78)	4.97 (0.34)	5.77 (0.5)	17.2	*	*	n.s.
FL_GM_	136.42 (13.55)	148.76 (14.05)	168.2 (13.67)	16.01	*	*	n.s.
Gyrus Rectus_GM_	3.05 (0.47)	3.49 (0.35)	3.97 (0.37)	15.79	*	*	*
IFG_GM_	18.37 (2.33)	20.35 (2.38)	23.32 (2.01)	14.25	*	*	n.s.
MOFG_GM_	9.6 (1.22)	10.64 (1.14)	11.59 (0.75)	10.17	*	n.s.	n.s.
PreG_GM_	19.74 (1.97)	20.93 (2.65)	23.65 (2.58)	8.19	*	n.s.	n.s.
MFG_GM_	36.52 (5.01)	39.58 (4.63)	43.24 (4)	6.32	*	n.s.	n.s.
Temporal Lobe							
TL_GM_	108.26 (5.81)	112.77 (12.12)	129.98 (9.88)	16.48	*	*	n.s.
STG_GM_	27.04 (1.57)	28.97 (4.1)	34.26 (3.54)	15.83	*	*	n.s.
ITG_GM_	23.22 (1.9)	24.21 (2.82)	27.52 (1.6)	11.83	*	*	n.s.
MTG_GM_	23.7 (1.82)	24.79 (3.52)	28.87 (2.5)	11.52	*	*	n.s.
Hippocampus & Amygdala	8.85 (0.91)	9.12 (1.83)	10.93 (1.2)	7.55	*	*	n.s.
PHG_GM_	9.7 (0.72)	9.92 (0.91)	11.0 (0.98)	7.41	*	*	n.s.
FG_GM_	15.75 (1.12)	15.76 (1.36)	17.39 (1.46)	5.92	*	*	n.s.

Caudate	6.37 (0.91)	7.42 (0.76)	8.29 (0.75)	16.97	*	n.s.	*
Insula	11.82 (0.83)	13.06 (1.61)	14.89 (1.5)	16.04	*	*	n.s.
**White Matter**							
Frontal Lobe							
IFG_WM_	13.57 (1.9)	14.35 (2.18)	16.52 (1.93)	6.62	*	n.s.	n.s.
LOFG_WM_	1.36 (0.4)	1.58 (0.38)	1.84 (0.27)	5.43	*	n.s.	n.s.
MOFG_WM_	4.32 (0.98)	4.8 (0.92)	5.41 (0.52)	4.84	*	n.s.	n.s.
MFG_WM_	41.48 (5.26)	45.47 (6.35)	48.01 (3.69)	4.76	*	n.s.	n.s.
Temporal Lobe							
MTG_WM_	20.98 (2.18)	22.23 (3.13)	24.53 (1.98)	5.98	*	*	n.s.

n.s.  =  not significant; *  =  p0.05, corrected for multiple comparisons

### Annualised brain volumetric changes

**Figure 2:**
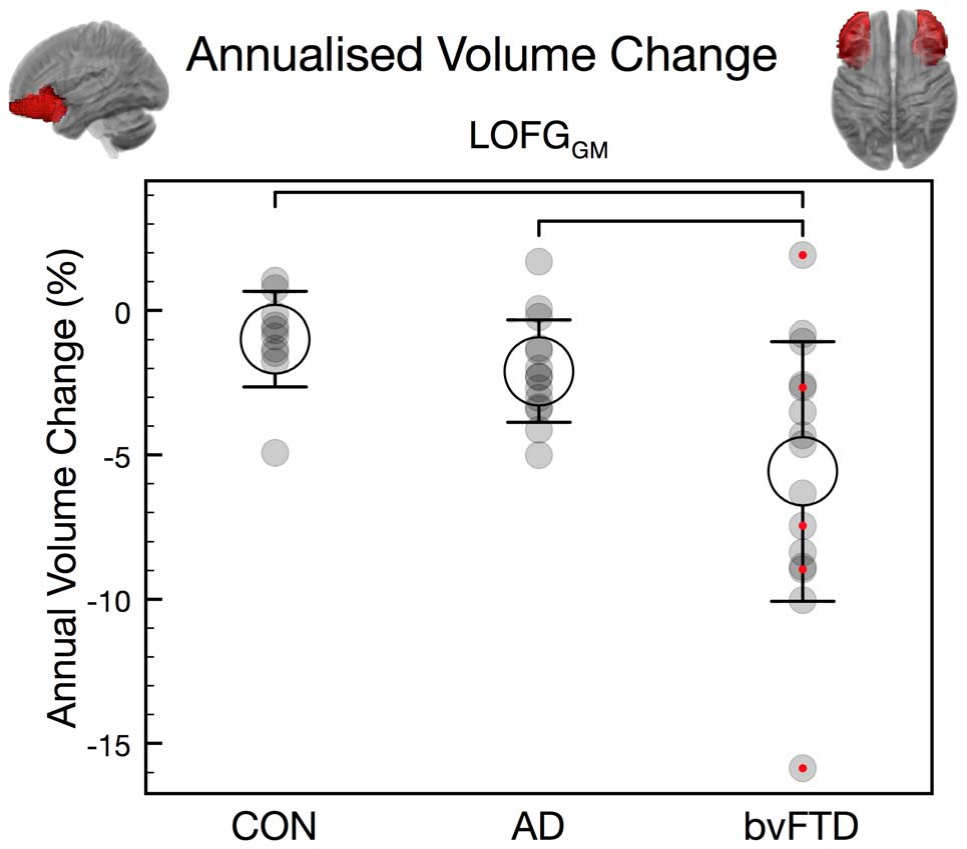
Annualised volume change (%; mean +/– 1 S.D.) of LOFG grey matter. **Brackets** indicate significant group differences. Red dots indicate data from Freiburg. CON  =  control participants.

The univariate analyses revealed a significant group effect for annualised change of several white matter areas (MTG_WM_, TL_WM_, ITG_WM_, FL_WM_, SFG_WM_, PreG_WM_, IFG_WM_, STG_WM_, MFG_WM_, MOFG_WM_, FG_WM_, PHG_WM_), and additional grey matter changes in the lateral orbitofrontal gyrus (LOFG_GM_), insula and the hippocampus & amydala (Table 4). Post-hoc tests revealed that the bvFTD group had significantly larger atrophy rates of each of these structures than the group of control participants, except for white matter in the fusiform gyrus (FG_WM_). AD patients, compared to controls, had larger atrophy rates of temporal lobe (TL_WM_) and middle temporal gyrus white matter (MTG_WM_). The bvFTD patients had larger atrophy rates than AD in the lateral orbitofrontal gyrus (LOFG_GM_) (Figure 2).

**Table 4:** Anatomical structures that showed significant group differences of annualised volume change (FDR-corrected p0.05). Per group mean and S.D. (in %), univariate between-subjects effects, and pairwise post-hoc Bonferroni test results.

Dependent Variable	bvFTD	AD	CON	F(2,36)	bvFTD vs CON	AD vs CON	bvFTD vs AD
**Grey Matter**							
Frontal Lobe							
LOFG_GM_	–5.57 (4.5)	–2.1 (1.77)	–0.99 (1.65)	7.74	*	n.s.	*
Temporal Lobe							
Hippocampus & Amygdala	–3.61 (3.24)	–3.09 (1.62)	–0.95 (0.78)	4.37	*	n.s.	n.s.

Insula	–3.59 (2.85)	–2.37 (1.25)	–0.73 (0.88)	6.27	*	n.s.	n.s.
**White Matter**							
Frontal Lobe							
FL_WM_	–3.7 (2.57)	–2.35 (1.21)	–0.7 (1.4)	7.58	*	n.s.	n.s.
SFG_WM_	–3.84 (2.97)	–2.56 (1.35)	–0.74 (1.49)	6.20	*	n.s.	n.s.
PreG_WM_	–3.24 (3.06)	–1.84 (2.63)	0.75 (2.82)	5.88	*	n.s.	n.s.
IFG_WM_	–4.35 (3.28)	–3.05 (1.55)	–1.26 (1.05)	5.37	*	n.s.	n.s.
MFG_WM_	–3.29 (2.7)	–2.02 (1.08)	–0.91 (1.02)	4.98	*	n.s.	n.s.
MOFG_WM_	–5.46 (2.72)	–3.46 (3.46)	–2.04 (1.46)	4.75	*	n.s.	n.s.
Temporal Lobe							
MTG_WM_	–2.64 (1.24)	–2.08 (0.97)	–0.76 (0.47)	10.83	*	*	n.s.
TL_WM_	–3.75 (2.03)	–2.85 (1.25)	–0.88 (0.86)	10.65	*	*	n.s.
ITG_WM_	–5.3 (3.29)	–3.87 (2.25)	–0.99 (1.3)	8.65	*	n.s.	n.s.
STG_WM_	–3.9 (2.91)	–3.43 (2.87)	–0.63 (1.72)	5.04	*	n.s.	n.s.
FG_WM_	–3.56 (3.26)	–1.77 (1.3)	–1.12 (0.77)	4.25	n.s.	n.s.	n.s.
PHG_WM_	–6.81 (6.79)	–5.59 (2.34)	–1.55 (1.35)	4.24	*	n.s.	n.s.

n.s.  =  not significant; *  =  p0.05, corrected for multiple comparisons.

The measures which showed a significant difference between bvFTD and AD in the former analyses (caudate and gyrus rectus_GM_ volumes at clinic presentation, and LOFG_GM_ atrophy rate) were subjected to a post-hoc binary logistic regression to determine the best predictor of diagnosis (bvFTD vs. AD). The forward stepwise logistic regression revealed that caudate volume at clinic presentation alone predicted 23/29 (79%) cases correctly (p<0.05; Nagelkerke's R^2^  =  0.40). When the gyrus rectus_GM_ was included in addition to caudate volume, the prediction improved to 24/29 (83%) (Nagelkerke's R^2^  =  0.57; p(Caudate) <0.05; p(gyrus rectus_GM_)  =  0.062). Atrophy rate of the LOFG_GM_ did not significantly contribute to the prediction.

### Subgroup analyses contrasting data from two scanning sites

In order to test for systematic differences between volumetric data acquired at the two different scanning sites, confirmatory T-tests were applied (p  =  0.05). No significant differences were observed between bvFTD patients from Sydney and bvFTD patients from Freiburg regarding caudate volume, gyrus rectus_GM_ volume, or volume change of LOFG_GM_. Furthermore, each of the two bvFTD subgroups showed a significant difference compared to CON concerning caudate volume, gyrus rectus_GM_ volume, and volume change of LOFG_GM_. Similarly, each of the two bvFTD subgroups showed a significant difference compared to AD regarding caudate volume, gyrus rectus_GM_ volume, and volume change of LOFG_GM_, with the exception of the comparison of gyrus rectus_GM_ volume between bvFTD (Freiburg) and AD, which failed to reach significance.

## Discussion

This study reports annual grey and white matter atrophy changes in bvFTD and AD. Our findings indicate that white matter changes are of particular importance when considering longitudinal neuroimaging changes in these neurodegenerative conditions.

### Cross-sectional findings

Atrophy of frontal, insular and temporal lobe structures was observed in both bvFTD and AD at clinic presentation, in line with previous reports in bvFTD [10], [12], [23]–[25] and AD [26]. The largest inter-group difference was observed in the grey matter volumes of the superior frontal gyrus (SFG_GM_) being smaller in both patient groups than controls. The latter region is commonly associated with executive functions such as planning and execution, is often reported to be atrophic in bvFTD [24], [25], [27], [28], and to a lesser extent in AD [26], [29]. The bvFTD group also showed marked caudate atrophy compared to controls [27], [30] and AD [31]. Caudate is, therefore, a potentially important imaging biomarker for bvFTD and is in keeping with its role in response inhibition [32], probabilistic learning [33] and stereotypical behaviour [34], the latter a hallmark feature of bvFTD. Atrophy of the ventromedial prefrontal cortex is recognised to be one of the earliest features of bvFTD [11]. Our findings of greater medial orbitofrontal cortex atrophy (gyrus rectus) in bvFTD than AD is, therefore, in line with previous reports [13], [23], [27]. Importantly, this region has been associated with disinhibition which is more common in bvFTD than in AD [4], [35]. Interestingly, none of the underlying white matter volumes in those regions distinguished between bvFTD and AD at presentation.

### Longitudinal findings

Both groups’ annual progression rates were highest in temporal white matter (temporal lobe, middle temporal gyrus, inferior temporal gyrus). In addition, the bvFTD group showed significant annual changes in grey matter regions, with particularly the lateral orbitofrontal cortex and insula being affected. Annual volume decrease in the group of healthy elderly controls was ∼1% overall which is commensurate with prior estimates [36], [37]. By contrast, the annual decrease was considerably elevated in AD (2.5% averaged over participants and structures) and in bvFTD (3.6% averaged over participants and structures) in line with previous reports of greater atrophy rates in FTD compared to AD [38]–[40].

Although the caudate displayed marked atrophy at clinic presentation in bvFTD, there was no significantly elevated atrophy rate over a 12 months period. By contrast, the LOFG_GM_ volume continued to decline at a high rate (grey matter volume decline of about 6% in bvFTD vs. 1% in controls). These findings highlight the variability of progression slopes between brain regions, which can be potentially used for future neuroimaging disease stageing. It is likely that atrophy in gyrus rectus and caudate, which are known to be affected early in bvFTD [11], [41], might have already plateaued by the time patients present with little further decline over the next 12 months. Of note was the finding that white matter atrophy progression exceeds grey matter degeneration in both diseases but particularly bvFTD. The fact that bvFTD patients show greater WM degeneration at presentation has been reported before [11], [27]. We extend these findings by showing for the first time that bvFTD patients had significantly greater changes over time (see Table 4).

### Clinical implications

The results highlight the fact that MRI volumetry may assist in the differential diagnosis of bvFTD and AD with loss of volume of the caudate supporting a clinical diagnosis of bvFTD. Identification of subcortical changes might therefore be more predictive than cortical changes, which often have been shown to overlap between the diseases. The observed longitudinal changes in our study may also be important for disease stageing of bvFTD and AD, in conjunction with other disease severity measures such as the FRS [18] or CDR-FTLD [42]. Finally, the longitudinal changes will allow tracking of disease progress tracking, which is critical to measuring the efficacy of disease modifying therapies. In addition to tracking overall volume loss it may be germane to consider particular structures such as temporal white matter or grey matter of the lateral orbitofrontal gyrus.

### Limitations & future directions

Despite these promising findings, our study had several limitations. We pre-selected regions, based on current knowledge of bvFTD, which potentially excluded other regions that might be of interest, e.g., parietal regions. Data from two different scanning sites have been pooled in the analyses. However, for each individual the scanning site and protocol have consistently been held constant. Moreover, subgroup analyses did not detect any systematic difference between data from the two sites, and subgroups essentially replicated the findings obtained from pooled data. Volumetric measures of each structure were combined over the left and right hemispheres in this study, which excludes analyses of atrophy asymmetries. The investigation of atrophy in degenerative diseases with a well-known lateralisation, like primary progressive aphasia, might benefit from separate assessment of left and right hemisphere structures. There was also no pathological confirmation of our cases, which leaves the possibility that a percentage of cases had a mis-match of clinical diagnosis and underlying pathology. Finally, surprisingly high atrophy progression rates were observed in the white matter of several brain structures, which suggests that longitudinal WM imaging with diffusion tensor imaging might provide additional insight into ongoing disease processes in bvFTD and AD.

## Conclusion

In summary, we show that automated MRI volumetry may help to discriminate dementia patients from controls and differentiate between dementia subtypes. While grey matter atrophy is already present at clinic presentation in bvFTD and AD, white matter atrophy follows with the steepest decline.
